# Factors associated with changes in opioid analgesic prescribing after opioid agonist treatment initiation: A nationwide registry cohort study

**DOI:** 10.1016/j.dadr.2026.100426

**Published:** 2026-03-10

**Authors:** Gabriela Rolova, Svetlana Skurtveit, Anne Bukten, Ingvild Odsbu, Desiree Eide

**Affiliations:** a Norwegian Centre for Addiction Research, University of Oslo, Postbox 1039, Blindern, Oslo 0315, Norway; b Norwegian Institute of Public Health, Postbox 222, Skøyen, Oslo 0213, Norway; c Department of Addictology, First Faculty of Medicine, Charles University, Apolinářská 4, Prague 2, 128 00, Czechia; d Department of Addictology, General University Hospital in Prague, Apolinářská 4, Prague 2, 128 00, Czechia

**Keywords:** Chronic pain, OMT, Opioid maintenance treatment, Substance use, Pain management, Prescription opioids

## Abstract

**Background:**

Pain is common among individuals receiving opioid agonist treatment (OAT). This study aims to investigate factors associated with changes in opioid analgesic dispensing among treatment-naïve patients initiating OAT.

**Methods:**

We analyzed nationwide health and dispensing records of patients initiating OAT (N = 3783) in Norway from 2014 to 2023. The cohort included new OAT patients who completed their first year of treatment. The primary outcomes were changes in opioid analgesic use during the year before and after OAT initiation. Logistic regression was used to identify factors associated with continued opioid analgesic use after initiating OAT.

**Results:**

Over one-third (n = 1329, 35%) of new OAT patients were prescribed opioid analgesics in the year prior to treatment initiation. Following OAT, there was a 37% reduction. Daily opioid amounts in morphine milligram equivalents decreased after OAT initiation across all four percentile strata. Continued opioid analgesic use after initiation of OAT was more likely among women (adjusted odds ratio [aOR] 1.32, 95% CI 1.04–1.68) and participants aged > 56 years (aOR 1.71, CI 1.07–2.72). Pain-related diagnoses (aOR 2.45, CI 1.93–3.11), depression or anxiety disorders (aOR 1.59, CI 1.23–2.03), and bipolar/schizophrenia disorders (aOR 1.89, CI 1.19–2.98) were associated with higher odds of continued opioid analgesic use.

**Conclusions:**

Opioid analgesic use was prevalent among OAT patients before treatment initiation, with reductions observed within one year. Continued opioid use among those with pain suggests that OAT alone may not fully address pain management needs.

## Highlights

• This study analyzed 3783 opioid agonist treatment (OAT) naïve patients.
• One-third of new OAT patients used opioid analgesics before treatment initiation.
• Approximately 20% continued to use opioid analgesics after starting treatment.
• Women and those over 56 were more likely to continue opioid use after OAT.
• Higher rate of continued opioid use for those with pain and psychiatric diagnoses.

## 1 Introduction

Opioid agonist treatment (OAT) is the cornerstone of care for opioid use disorder (OUD) and has been shown to reduce illicit opioid use, overdose mortality, and all-cause mortality (Clausen et al., 2008, Santo et al., 2021, Sordo et al., 2017). However, many patients entering OAT have concurrent acute or chronic pain conditions, for which opioid analgesics may be prescribed before or during treatment (Cragg et al., 2019, Eyler, 2013). The management of pain in individuals receiving OAT is clinically complex, given altered opioid tolerance, concerns about misuse, and uncertainty among prescribers regarding the appropriateness of co-prescribing opioid analgesics (De Aquino et al., 2021, Guichard et al., 2022). Prior studies suggest that opioid prescribing patterns may change following OAT initiation (Ellis et al., 2021), but population-level evidence describing the extent, magnitude, and predictors of these changes remains limited.

Understanding how opioid analgesic use evolves before and after OAT initiation is essential to inform pain management strategies and to identify subgroups at risk for continued opioid exposure during treatment. Therefore, the primary objective of this study was to analyze changes in opioid analgesic prescribing among a treatment-naïve cohort of OAT patients. Specifically, we aimed to identify shifts in prescribing patterns before and after the start of OAT and to investigate factors associated with continued opioid analgesic use one year after OAT initiation.

## 2 Methods

### 2.1 Study design

This registry-based cohort study used nationwide, linked health and dispensing records of patients initiating OAT in Norway between 2014 and 2023.

### 2.2 Setting

Norway has a tax-based healthcare system that provides universal coverage to all citizens and legal residents. Opioid analgesics are prescription-only and may be fully or partially reimbursed for patients in palliative care or with chronic pain, respectively.

Introduced nationally in 1998, the Norwegian OAT program gradually adopted a low-threshold, harm reduction-oriented approach, which eventually emphasized long-term duration and rarely discharged patients for ongoing illicit drug use (Waal, 2007). The program is publicly funded and offered free or at low cost to all patients. OAT is initiated in specialist care and often later transferred to primary care. Treatment retention is relatively high, with 73% of patients remaining in OAT after one year (Bukten et al., 2014).

OAT medications are administered under daily supervision or provided as take-home prescriptions for up to two weeks, depending on patient clinical stability. OAT daily dosages in Norway exceed international norms, with 2019 averages of 91 mg methadone, 14.6 mg buprenorphine, and 12.8 mg buprenorphine-naloxone (Lobmaier et al., 2021).

### 2.3 Data sources

We used data from the POINT-study including several national health and population registries covering the period 2010–2023 (Bakken et al., 2020, Hamina et al., 2022). Individual-level patient records were linked through pseudonymized unique personal identification number assigned to all Norwegian residents.

Individuals receiving OAT and information on the use of opioid analgesics were obtained from the Norwegian Prescribed Drug Registry (NorPD). This registry contains data on all drugs dispensed from Norwegian pharmacies, which are legally required to report data electronically. It includes information on the dispensed drug, date of dispensation, volume in Defined Daily Doses (DDD), and administration form, identified via Nordic Article Numbers specific to individual products. NorPD uses the Anatomical Therapeutic Chemical/Defined Daily Doses (ATC/DDD) classification system (WHO Collaborating Centre for Drug Statistics Methodology, 2025).

The Norwegian Patient Registry (NPR), which contains specialized outpatient healthcare episodes and hospitalization records, was used to retrieve information on OUD and OAT. Information on comorbidities was obtained from the NPR and the Norwegian Registry of Primary Health Care, including date of diagnosis and the respective ICD-10 codes (Supplementary Table 1). Diagnoses received in the secondary healthcare are recorded according to ICD-10, and in primary healthcare according to the International Classification of Primary Care, 2nd edition (ICPC-2).

The Cause of Death Registry was used to identify individuals who died within 365 days of their first dispensed OAT medication (i.e., treatment initiation).

Socio-demographic characteristics were taken from Statistics Norway.

### 2.4 Study population and study period

The study population included all patients who filled at least one prescription for an OAT medication, i.e., methadone (ATC code N07BC02), buprenorphine (N07BC01), levomethadone (N07BC05), and buprenorphine–naloxone (N07BC51). These formulations are almost exclusively used for treating OUD in Norway.

Between 2010 and 2022, a total of 14,753 individuals were dispensed at least one OAT medication. Of these, we identified 12,868 with a registered OUD diagnosis (ICD-10 codes F11.2 or Z50.3). To identify new OAT patients, we excluded those whose first dispensation occurred between 2010 and 2013 (n = 8665), resulting in 3853 individuals who initiated treatment between 2014 and 2022. An additional 70 patients who died within the first year of OAT initiation were excluded.

The final study population comprised 3783 individuals who initiated OAT during 1 January 2014 and 31 December 2022 and survived at least one-year post-initiation.

### 2.5 Measures

The main outcomes were changes in opioid analgesic use during the 365 days before and after OAT initiation, and factors associated with continued use, post-OAT initiation. In addition, we examined the proportion of people using new vs. existing prescription opioids after OAT initiation, along with pre-post changes in daily volumes dispensed and use of “weak” and “strong” opioids.

Opioid analgesics were defined as all substances in ATC group N02A. “Weak” opioids included codeine combinations (N02AJ06, N02AJ07) and tramadol, either alone (N02AX02) or in combination (N02AJ13); all other N02A opioids were classified as “strong”. To account for differences in opioid potencies, we calculated morphine milligram equivalents (MMEs) by multiplying dispensed DDDs by substance- and administration form-specific conversion factors (Rolova et al., 2025).

Pain-related diagnosis, Depression/anxiety disorder, bipolar disorder/schizophrenia and Attention-deficit/Hyperactivity disorder (ADHD) were defined according to ICD-10 and ICPC-2 codes (Supplementary Table 1). Comorbidities were measured during the first year since OAT admission.

### 2.6 Statistical analysis

We present descriptive statistics as frequencies, means with standard deviations (SDs), and medians with interquartile ranges (IQRs), stratified by sex.

One-year prevalence of opioid analgesic dispensation was calculated as the proportion of individuals who filled at least one prescription in the 365 days before or after OAT initiation. Although the data reflects dispensations, we employ the term ‘use’ as a proxy and apply this terminology throughout the manuscript.

Logistic regression was used to assess the association between demographic and clinical covariates prior to OAT initiation and continued opioid analgesic use after OAT initiation. We report results as unadjusted and adjusted odds ratios (ORs and aORs) with 95% confidence intervals (CIs).

Statistical analyses were performed using IBM SPSS Statistics (version 27; Armonk, NY).

### 2.7 Ethics

The research protocol was approved by the Regional Committee for Medical and Health Research (registration number 2019/656/REC South-East C).

## 3 Results

Of the 3783 individuals included in the study, 69.5% were men, the mean age was 37.8 years, and 47.7% received buprenorphine (Table 1). Most of the cohort was single, unemployed, and had lower secondary education or less; depression/anxiety disorders were common (31.3%), and 26.9% had a pain-related diagnosis.

**Table 1:** Characteristics of a cohort of opioid agonist treatment (OAT) naïve individuals and the use of opioid analgesics one-year pre-and post- OAT initiation.

	**Women**		**Men**		**Total**	
	**(N = 1154)**		**(N = 2629)**		**(N = 3783)**	
**N of individuals, n (%)**	1154 (30.5)		2629 (69.5)		3783	
**Age at OAT initiation, years – mean (median)**	37.5 (35)		37.8 (36)		37.8 (36)	
**OAT opioid at initiation, n (%)**						
Buprenorphine	531 (46.0)		1274 (48.5)		1805 (47.7)	
Methadone*	216 (18.7)		367 (14.0)		583 (15.4)	
Buprenorphine-naloxone	407 (35.3)		988 (37.6)		1395 (36.9)	
**Educational attainment, n (%)**						
Lower secondary school or less	794 (68.8)		1934 (73.6)		2728 (72.1)	
Upper secondary school	265 (23.0)		559 (21.3)		824 (21.8)	
Higher education	95 (8.2)		136 (5.2)		231 (6.1)	
**Marital status, n (%)**						
Single	1045 (90.6)		2477 (94.2)		3522 (9.1)	
Married/with partner	85 (7.4)		142 (5.4)		227 (6.0)	
Widowed	24 (2.1)		10 (0.4)		34 (0.9)	
**Employment status, n (%)**						
Employed	77 (6.7)		245 (9.3)		322 (8.5)	
Not employed	1077 (93.3)		2384 (90.7)		3461 (91.5)	
**Pain-related diagnosis**	412 (35.7)		604 (23.0)		1016 (26.9)	
**Depression/anxiety disorder, n (%)**	409 (35.4)		775 (29.5)		1184 (31.3)	
**Bipolar disorder/schizophrenia, n (%)**	85 (7.4)		213 (8.1)		298 (7.9)	
**ADHD, n (%)**	130 (11.3)		306 (11.6)		436 (11.5)	
	**1-year pre-OAT**	**1-year post-OAT**	**1-year pre-OAT**	**1-year post-OAT**	**1-year pre-OAT**	**1-year post-OAT**
**Age group at OAT initiation, n (%)**						
17–30	111 (29.8)	71 (19.1)	182 (25.0)	131 (18.0)	293 (26.7)	202 (18.4)
31–40	146 (39.8)	92 (25.1)	266 (28.1)	173 (18.3)	412 (31.4)	265 (20.2)
41–55	186 (56.2)	114 (34.4)	304 (38.2)	170 (21.4)	490 (43.5)	284 (25.2)
56 +	58 (69.0)	37 (44.0)	76 (46.9)	52 (32.1)	134 (54.5)	89 (36.2)
**Any opioid analgesic dispensation, n (%)**	501 (43.4)	314 (27.2)	828 (31.5)	526 (20.0)	1329 (35.1)	840 (22.2)
**MMEs per day of opioid analgesics – percentiles**						
25th percentile	0,8	0,4	0,7	0,4	0,7	0,4
50th percentile	6,5	2,5	5,3	1,6	5,7	1,9
75th percentile	55,2	12,3	36,8	9,2	42,9	10,4
90th percentile	194,7	79,4	173	40,9	179,6	52,3
**"Weak" opioid analgesics, n (%)**	381 (33.0)	213 (18.5)	598 (22.7)	367 (14.0)	979 (25.9)	580 (15.3)
**"Strong" opioid analgesics, n (%)**	239 (20.7)	145 (12.6)	386 (14.7)	223 (8.5)	625 (16.5)	369 (9.8)

OAT =  opioid agonist treatment; MME =  morphine milligram equivalent, ADHD =  attention deficit/hyperactivity disorder

* Including the use of levomethadone (n = 7)

** percentiles listed as 25%, 50%, 75%, 90%;

Notably, over one-third of new OAT patients were dispensed an opioid analgesic in the year preceding their treatment initiation (n = 1329, 35.1%). Among these patients, women—particularly those in the oldest age group (56 years and older)—exhibited the highest rates of pre-OAT opioid analgesic dispensing, with 69.0% (n = 58) in this demographic.

One year after starting OAT, there were 840 participants (22.2%) that had opioid analgesics dispensed. Women accounted for a larger proportion of individuals receiving opioids after starting OAT, with 27.2% compared to 20.0% for men (Table 1).

Daily opioid amounts in MMEs decreased after OAT initiation across all four percentile strata. This decrease was particularly pronounced in the third percentile group, whose MME dropped by 75.8% during the pre- and post-OAT period (from 42.9 to 10.4). Furthermore, the prescribing of strong opioid analgesics decreased in the post-OAT period, with less than 10% of patients receiving a strong opioid.

Women were more likely to continue opioid analgesic use after starting OAT (adjusted odds ratio [aOR] 1.32, CI: 1.04–1.68) (Table 2). We observed that increasing age was associated with increasingly higher odds of continued opioid analgesic use, as those aged over 56 years had the highest odds (aOR 1.71, CI: 1.07–2.72). Further, the higher the MME, the higher the risk for continued use (aOR 1.01, CI: 1.00–1.01). Receiving buprenorphine-naloxone was associated with decreased odds (aOR 0.71, CI: 0.55–0.93). Regarding comorbidities, a pain-related diagnosis was associated with increased odds of continued opioid analgesic use (aOR 2.45, CI: 1.93–3.11), as were depression/anxiety disorders (aOR 1.59, CI: 1.23–2.03) and bipolar/schizophrenia disorders (aOR 1.89, CI: 1.19–2.98).

**Table 2:** Factors associated with continued use of opioid analgesics after opioid agonist treatment initiation.

	**Pre-OAT use of opioid analgesics (N = 1329)**			
	**Crude OR**	**Adjusted OR**	**95% CI**	**p-value**
**Gender**				
Men	1.0 (ref)	1.0 (ref)		
Women	1.54	1.32	1.04–1.68	0.024
**Age groups, years**				
17–30	1.0 (ref)	1.0 (ref)		
31–40	1.32	1.26	0.91–1.76	ns
41–55	1.57	1.35	0.87–1.87	ns
56 +	2.28	1.71	1.07–2.72	0.024
**MMEs per day of opioid analgesics**	1.01	1.01	1.00–1.01	0.026
**OAT opioid at initiation**				
Buprenorphine	1.0 (ref)	1.0 (ref)		
Methadone*	1.23	0.91	0.66–1.26	ns
Buprenorphine-naloxone	0.70	0.71	0.55–0.93	0.013
**Pain-related diagnosis**				
No	1.0 (ref)	1.0 (ref)		
Yes	2.83	2.45	1.93–3.11	< 0.001
**Depression/anxiety disorder**				
No	1.0 (ref)	1.0 (ref)		
Yes	1.56	1.59	1.23–2.03	< 0.001
**Bipolar disorder/schizophrenia**				
No	1.0 (ref)	1.0 (ref)		
Yes	1.55	1.89	1.19–2.98	0.007
**Attention-deficit/Hyperactivity disorder (ADHD)**				
No	1.0 (ref)	1.0 (ref)		
Yes	0.92	0.94	0.64–1.38	ns
**Educational attainment**				
Lower secondary school or less	1.0 (ref)			
Upper secondary school	0.79	0.80	0.50–1.27	ns
Higher education	0.58	0.74	0.48–1.15	ns
**Marital status**				
Single	1.0 (ref)			
Married/with partner	1.62	1.11	0.75–1.64	ns
Widowed	1.84	1.10	0.44–2.79	ns
**Employment status**				
Employed	1.0 (ref)			
Not employed	0.94	1.00	0.67–1.50	ns

⁎ Including levomethadone (n = 7); ns=  not significant; OAT =  opioid agonist treatment; OR =  odds ratio; CI =  confidence interval; ref =  reference group, MME =  morphine milligram equivalent

## 4 Discussion

This study found that opioid analgesic use was prevalent among OAT patients before treatment initiation, with reductions observed within one year after starting treatment. Women, individuals aged over 56 years, and those with pain-related or psychiatric diagnoses were more likely to continue opioid analgesic use after starting OAT. The findings from this study contribute to understanding the complexity of opioid prescribing and OAT.

Given that this study was an observational study of dispensing records, causal inferences about the effects of OAT on pain or prescribing cannot be drawn. The observed reduction in opioid prescribing following OAT may reflect one or more processes, such as a change in clinical need for opioid analgesics related to improved stabilization (Aas et al., 2020). Alternatively the reduction could reflect decreased analgesic effectiveness due to opioid tolerance (De Aquino et al., 2021). Further, the reduction could indicate insufficient pain management while in treatment. Unmanaged pain while in OAT can be highly problematic, with pain being found to be a primary factor leading to relapse (Ellis et al., 2021). This study does not allow the ability to distinguish among these mechanisms, and the relative contribution of each may differ across individuals and subgroups.

The higher likelihood of continued opioid dispensing among women and older adults is consistent with literature showing greater reported pain and different analgesic prescribing patterns in these groups (Barbosa-Leiker et al., 2020, Goetz et al., 2021). These associations may reflect underlying differences in healthcare utilization or unmeasured socioeconomic factors. Pain-related diagnoses were strongly associated with continued prescription opioid use after OAT, suggesting appropriate targeting but also that OAT alone may not fully address pain. Depression/anxiety, and bipolar/schizophrenia were also associated, highlighting the need to address both psychiatric comorbidities and pain management in OAT.

### 4.1 Strengths and limitations

This study's key strength is its use of a nationwide cohort for an unbiased examination of prescribing practices. By utilizing prescription registry data, it provides reliable insights into medication use among a hard-to-reach patient group, overcoming the recall bias common in traditional surveys. This data allows for consistent recording of prescriptions before and during treatment and offers a more efficient alternative to the resource-intensive recruitment of treatment-naïve patients. The national registry allows for comparisons of medication use across clinical populations and the general population.

However, the study has limitations. A primary concern is the inability to determine whether dispensed prescription opioids were actually used, raising questions about the data's practical implications. The lack of information on the clinical indications for prescription limits understanding of the context of opioid use. These limitations caution against over interpretation and identify areas for further research.

## 5 Conclusion

Our findings suggest that OAT may provide a stabilizing effect, reducing the need for opioid analgesics, while also raising concerns about potential insufficient management of chronic pain during OAT. Continued opioid use among those with pain suggests that OAT alone may not fully address pain management needs. While the mechanisms behind the reduction in opioid analgesic use cannot be determined from this study, the findings support the need for attention to pain management and mental health disorders among OUD patients, particularly among women.

## CRediT authorship contribution statement

**Desiree Eide:** Writing – review & editing, Writing – original draft, Supervision, Conceptualization. **Gabriela Rolova:** Writing – review & editing, Writing – original draft, Formal analysis, Conceptualization. **Ingvild Odsbu:** Writing – review & editing, Writing – original draft, Project administration, Data curation, Conceptualization. **Anne Bukten:** Writing – review & editing, Writing – original draft, Conceptualization. **Svetlana Skurtveit:** Writing – review & editing, Writing – original draft, Project administration, Methodology, Funding acquisition, Formal analysis, Data curation, Conceptualization.

## Funding

SS and IO were partially funded by the 10.13039/501100005416Norwegian Research Council, Grant/Award Number: 320360; Johannes Amos Comenius Programme (OP JAK) – MSCA Fellowship CZ – UK3, [no. CZ.02.01.01/00/22_010/0008820] awarded to the author GR; Charles University institutional support program Cooperation (research area HEAS).

## Declaration of Competing Interest

None

## Footnotes

Appendix A Supplementary data associated with this article can be found in the online version at doi:10.1016/j.dadr.2026.100426.
