# Supplementary material for: Factors associated with changes in opioid analgesic prescribing after opioid agonist treatment initiation: A nationwide registry cohort study
Source: Drug Alcohol Depend Rep. 2026 Mar 10;19:100426. doi: 10.1016/j.dadr.2026.100426 (PMC13014969; doi:10.1016/j.dadr.2026.100426)
Supplement: Supplementary file 1 — Supplementary material [file mmc1.docx]

| **Supplementary Table 1. Description of clinical covariates** | | | |
| --- | --- | --- | --- |
|  | | | |
| **Variable name** | **Data source** | **Coding system** | **Definition** |
| **Pain-related diagnosis** | | | |
| Back pain | NPR | ICD-10; | M50, M51, M53, M54; |
|  | KUHR | ICPC-2 | L02, L03, L83, L84, L86 |
| Osteoarthritis | NPR | ICD-10; | M15–M19; |
|  | KUHR | ICPC-2 | L89–L91 |
| Arthritis | NPR | ICD-10; | M05-M14 |
|  | KUHR | ICPC-2 | L88 |
| Migraine and headaches | NPR | ICD-10; | G43-G44, R51 |
|  | KUHR | ICPC-2 | N01, N89, N90, N95 |
| Neuropathic pain | NPR | ICD-10; | G50, G53-G59, G60-G64, M79.2 , S14, S24, S34, S44, S74 |
|  | KUHR | ICPC-2 | N92, N94, S70 , N81 |
| Abdominal and pelvic pain | NPR | ICD-10; | R10, K50-K51, N80 |
|  | KUHR | ICPC-2 | D01, D02, D06, D93-D94, X99 |
| Other pain | NPR | ICD-10; | M79.7, M80, M84, G89, R52 |
|  | KUHR | ICPC-2 | L95, L99, L18, A01 |
| **Depression/anxiety disorder** | | | |
| Depressive disorders | NPR | ICD-10; | F32–F34, F39; |
|  | KUHR | ICPC-2 | P76 |
| Phobias and anxiety disorders | NPR | ICD-10; | F40–F44; |
|  | KUHR | ICPC-2 | P74, P79 |
| **Bipolar disorder/Schizophrenia** | | | |
| Bipolar disorder | NPR | ICD-10; | F30, F31 |
|  | KUHR | ICPC-2 | P73 |
| Schizophrenia and related disorders | NPR | ICD-10; | F20-F29 |
|  | KUHR | ICPC-2 | P72 |
| **Attention-deficit/Hyperactivity disorder (ADHD)** | NPR | ICD-10; | F90 |
|  | KUHR | ICPC-2 | P81 |
| NPR = Norwegian Patient Registry; KUHR = Norwegian Registry for Primary Health Care | | | |
| ICD-10 = International Classification of Diseases, 10th revision; ICPC-2 = International Classification of Primary Care, 2nd revision | | | |
